# Chemical composition and biological activity of *Peucedanum dhana* A. Ham essential oil

**DOI:** 10.1038/s41598-021-98717-y

**Published:** 2021-09-27

**Authors:** Sarunpron Khruengsai, Teerapong Sripahco, Narawadee Rujanapun, Rawiwan Charoensup, Patcharee Pripdeevech

**Affiliations:** 1 School of Science, Mae Fah Luang University, Chiang Rai, 57100 Thailand; 2 Medicinal Plants Innovation Center of Mae Fah Luang University, Chiang Rai, 57100 Thailand; 3 School of Integrative Medicine, Mae Fah Luang University, Chiang Rai, 57100 Thailand; 4 Center of Chemical Innovation for Sustainability (CIS), Mae Fah Luang University, Chiang Rai, 57100 Thailand

**Keywords:** Biological techniques, Chemical biology, Plant sciences

## Abstract

The essential oil was extracted from *Peucedanum dhana* A. Ham, which grows in Thailand, using a Clevenger apparatus, resulting in an oil yield of 0.76% w/w. Forty-two compounds were identified using gas chromatography-mass spectrometry. The major compounds were *trans*-piperitol (51.23%), β-pinene (11.72%), o-cymene (11.12%), γ-terpinene (9.21%), and limonene (4.91%). The antimicrobial activity of the *P. dhana* essential oil was investigated by measuring the inhibition zone diameter, minimum inhibitory concentration (MIC), and minimum microbicidal concentration (MMC). The inhibition zone diameters of *P. dhana* essential oil (1000 µg/mL) against tested pathogens ranged from 10.70 to 40.80 mm. Significant antimicrobial activity against tested pathogens was obtained, with MIC and MMC values of 62.50–250 µg/mL and 250–1000 µg/mL, respectively. *Escherichia coli*, *Pseudomonas aeruginosa,* and *Enterobacter aerogenes* exposed to *P. dhana* essential oil at the MIC were analysed by flow cytometry using propidium iodide (PI) and SYTO9 to assess membrane integrity compared to *trans*-piperitol and β-pinene. After 24 h, treatments with *trans*-piperitol resulted in the most significant cell membrane alteration and depolarization followed by *P. dhana* essential oil and β-pinene, respectively. It was demonstrated that the *P. dhana* essential oil presented antibacterial action against *E. coli*, *P. aeruginosa,* and *E. aerogenes*. The antioxidant activity of *P. dhana* essential oil was measured using 2,2-diphenyl-2-picrylhydrazyl (DPPH) and 2,2-azinobis(3-ethylbenzothiazoline-6-sulfonic acid) diammonium (ABTS) scavenging activity assays. The IC_50_ values obtained from the DPPH and ABTS methods were 9.13 and 9.36 mg/mL, respectively. The cytotoxic effect of *P. dhana* oil was tested against human colonic adenocarcinoma (SW480), human lung adenocarcinoma (A549), cervical cancer (Hela), and murine fibroblast (3T3L1) cells using the 3-(4,5-dimethylthiazol-2-yl)-2,5-diphenyltetrazolium bromide (MTT) assay. The essential oil had cytotoxicity against all cancer cells, with significant cytotoxicity towards SW480 cells. As a control experiment, two pure compounds—*trans*-piperitol and β-pinene, were also tested for their antimicrobial, antioxidant, and cytotoxic activity. Both compounds showed varied activity in all assays. The results indicate that *P. dhana* essential oil could be used as a source of functional ingredients in food and pharmaceutical applications.

## Introduction

The genus *Peucedanum* belongs to the Apiaceae family and is composed of more than 120 species^1^. Most species are widely cultivated in Europe, Asia, and Africa^1^. According to the ethnopharmacological history of this genera, several species were widely used as traditional medicines for cough, sore throat, cold, headache, angina, asthma, cramps, epilepsy, gastrointestinal disorders, rheumatism, gout, and cardiovascular disease^2^. Some species were also used as chemopreventive and antifebrile agents^1^. Moreover, some species were reported to possess antimicrobial, antifungal, antioxidant, anti-inflammatory, and anticancer properties^1^. Previous phytochemical studies on *Peucedanum* indicated the presence of several bioactive compounds such as coumarins, polyphenols, amines, glycosides, flavonoids, phenolic acids, and terpenes^1^.

Various *Peucedanum* species produced essential oils that were reported as expectorants, sedatives, diuretics, diaphoretics, and stomachic agents^1^. Their essential oils also displayed biological and pharmacological activities^1^. The essential oils of different *Peucedanum* species are composed of monoterpenes and sesquiterpene hydrocarbons, oxygenated sesquiterpenes, aliphatic alcohols, and esters^1^. For example, α-pinene (4.0–38.7%) was the major compound in essential oils from *P. officinale*, *P. alsaticum*, *P. austriacum*, *P. oreoselinum*, *P. longifolium*, and *P. cervaria*^3^. Limonene (44.1–82.4%) and α-pinene (4.0–11%) were the major compounds from *P. oreoselinum* essential oil^4^, while other monoterpenes or sesquiterpenes were reported in other species such as *P. ruthenicum* M. Bieb. and *P. paniculatum*^1^.

The *P. dhana* A. Ham is a plant species in the *Peucedanum* genus and is considered a rare herb^1,2,5–7^. It is a glabrous perennial tree with small yellow flowers and is calyx-teeth obsolete. It is beige with short or long hairs on the surface and a waxy coating. This plant was reported to have high medicinal value. Its roots were used as a tonic that promotes sexual desire^8^ and its fruits produce essential oils^9^. Although essential oils from other species of the *Peucedanum* genus are well studied, the chemical composition and the antimicrobial and antioxidant activities of essential oils from *P. dhana* are rarely explored. Therefore, the present study aims to investigate the chemical composition of essential oil extracted from *P. dhana* fruits and to evaluate the antimicrobial, antioxidant, and cytotoxic activities as a function of the composition.

## Results

### Chemical composition of P. dhana essential oil

The essential oil of *P. dhana* fruits was obtained by hydrodistillation with a yield of 0.76% w/w based on the dry weight. As presented in Table 1, 42 volatile compounds were identified, which represent 99.87% of the oil. *Trans*-piperitol, β-pinene, and o-cymene were the major volatile compounds of the *P. dhana* essential oil. These results showed there are over 98.2% monoterpene hydrocarbons in *P. dhana* essential oil.

**Table 1:** Volatile compounds of *P. dhana* essential oil by GC–MS.

Compound	DB1		DB5		% area
	RI^a^	RI^b^	RI^a^	RI^b^	
Heptanal	887	880	911	901	t^c^
Tricyclene	928	921	928	921	t
Artemisia triene			931	923	t
α-Thujene	932	925	934	924	t
α-Pinene	941	934	940	932	0.91 ± 0.41
Camphene	954	947	954	946	0.11 ± 0.21
Benzadehyde	943	936	966	952	t
Sabinene	974	967	979	969	0.11 ± 0.10
β-Pinene	980	973	983	979	11.72 ± 0.41
*cis*-meta-mentha-2,8-diene			991	987	t
Myrcene	990	983	996	990	0.22 ± 0.13
Dehydroxy-*trans*-linalool oxide			1002	992	t
Yomogi alcohol	994	987	1007	999	1.34 ± 0.22
δ-2-Carene	1014	1007	1014	1001	t
α-Terpinene	1017	1010	1020	1014	t
o-Cymene	1019	1012	1028	1022	11.12 ± 0.62
Limonene	1030	1023	1031	1024	4.91 ± 0.44
γ-Terpinene	1057	1050	1061	1054	9.21 ± 0.33
*trans*-Arbusculone			1073	1066	2.44 ± 0.36
Terpinolene	1086	1079	1091	1086	t
Linalool	1093	1086	1103	1095	0.12 ± 0.11
Pinocarvone	1147	1140	1161	1160	t
Borneol	1160	1153	1167	1165	t
Neoiso-isopulegol			1168	1167	t
Terpinen-4-ol	1171	1164	1179	1174	0.11 ± 0.11
α-Terpineol	1182	1175	1193	1186	0.14 ± 0.11
*cis*-4-Caranone			1200	1200	2.14 ± 0.41
Decanal	1192	1185	1207	1201	0.24 ± 0.31
*trans*-Piperitol	1200	1193	1217	1207	51.23 ± 0.42
Isobornyl acetate	1278	1271	1285	1283	1.33 ± 0.21
β-Cubebene	1359	1352	1390	1388	1.55 ± 0.13
Decyl acetate	1399	1392	1410	1407	0.51 ± 0.11
E-Caryophyllene	1414	1407	1417	1417	0.11 ± 0.10
δ-Amorphene	1473	1466	1519	1511	0.21 ± 0.10
α-Calacorene	1537	1530	1539	1539	t
Maaliol			1578	1566	t
2-Ethylbutyric acid, octyl ester			1581	1581	0.82 ± 0.25
*cis*-Dihydro-mayurone			1604	1595	t
guaiol	1595	1588	1615	1600	t
Muurola-4,10(14)-dien-1-β-ol			1635	1630	t
α-Muurolol	1633	1626	1650	1643	t
Pogostol			1657	1651	0.44 ± 0.21
Total					99.87 ± 0.26

^a^Calculated retention indices.

^b^Retention indices on DB-1 and DB-5 columns from literature^10,11^.

^c^Trace amount < 0.05.

### Antimicrobial activity

The antimicrobial activity of *P. dhana* essential oil was evaluated against seven pathogenic microorganisms, using disc diffusion and broth microdilution methods. The zone of inhibition diameter, MIC, and MMC values of the *P. dhana* essential oil and chloramphenicol (control) for the tested microorganisms are shown in Table 2. The zone of inhibition diameter of the *P. dhana* essential oil for all microbial strains was similar to those obtained from the positive control. The MIC for the bacterial pathogens ranged from 62.50 to 250 µg/mL, whereas the MIC for fungus was 125 µg/mL. The data obtained from the disc diffusion method indicated that *P. aeruginosa* ATCC 27853 was the most sensitive (inhibition zone diameter of 40.80 mm) to 1000 µg/mL of *P. dhana* essential oil compared to other tested pathogens, which ranged from 10.70 to 19.60 mm. *P. dhana* essential oil had the lowest MIC value (62.50 µg/mL) for all Gram-negative bacterial pathogens and high MIC values for Gram-positive bacteria and fungus, which ranged from 125 to 500 µg/mL. The lowest MMC value was 250 µg/mL for *P. aeruginosa* ATCC 27853, while the highest MMC value was 1000 µg/mL for *S. aureus* ATCC 25923. The antimicrobial activity of *trans*-piperitol and β-pinene pure compounds is depicted in Table 3. The zone diameter against the pathogens was 13.30–26.56 mm and 8.62–9.39 mm, for 1000 µg/mL of *trans*-piperitol and β-pinene, respectively. *Trans*-piperitol showed lower MIC and MMC values than those obtained from β-pinene. The results showed that the tested pathogens were more susceptible to *trans*-piperitol than β-pinene.

**Table 2:** Antimicrobial activity of *P. dhana* essential oil and chloramphenicol.

Microorganism	Chloramphenicol			*P. dhana* essential oil		
	MIC (µg/mL)	MMC (µg/mL)	Zone of inhibition diameter (mm) (1000 µg/mL)	MIC (µg/mL)	MMC (µg/mL)	Zone of inhibition diameter (mm) (1000 µg/mL)
**Gram-positive bacteria**						
*S. aureus* ATCC 25923	125.00^c^	500.00^b^	19.10 ± 0.01^c^	250.00^c^	1000.00^c^	18.20 ± 0.20^b^
*S. epidermidis* ATCC 12228	125.00^c^	500.00^b^	19.50 ± 0.01^c^	125.00^b^	500.00^b^	19.25 ± 0.25^b^
*B. subtilis* ATCC 6051	62.50^b^	500.00^b^	20.40 ± 0.01^b^	125.00^b^	1000.00^c^	19.60 ± 0.31^b^
**Gram-negative bacteria**						
*E. coli* ATCC 25922	62.50^b^	500.00^b^	10.10 ± 0.00^e^	62.50^a^	500.00^b^	12.90 ± 0.20^c^
*P. aeruginosa* ATCC 27853	31.25^a^	250.00^a^	40.30 ± 0.01^a^	62.50^a^	250.00^a^	40.80 ± 0.20^a^
*E. aerogenes* ATCC 13048	62.50^b^	500.00^b^	19.20 ± 0.03^c^	62.50^a^	500.00^b^	18.70 ± 0.30^b^
**Fungus**						
*C. albicans* ATCC 10231	62.50^b^	500.00^b^	17.50 ± 0.20^d^	125.00^b^	500.00^b^	10.70 ± 0.20^d^

The data are mean ± standard deviation. Different letters indicate significant differences (p < 0.05).

**Table 3:** Antimicrobial activity of *trans*-piperitol and β-pinene.

Microorganism	*trans*-Piperitol			β-Pinene		
	MIC (µg/mL)	MMC (µg/mL)	Zone of inhibition diameter (mm) (1000 µg/mL)	MIC (µg/mL)	MMC (µg/mL)	Zone of inhibition diameter (mm) (1000 µg/mL)
**Gram-positive bacteria**						
*S. aureus* ATCC 25923	125.00^c^	1000.00^d^	13.30 ± 0.21^f^	500.00^b^	1000.00^b^	8.86 ± 0.18^b^
*S. epidermidis* ATCC 12228	125.00^c^	500.00^c^	15.43 ± 0.22^e^	500.00^b^	1000.00^b^	8.78 ± 0.21^c^
*B. subtilis* ATCC 6051	125.00^c^	1000.00^d^	15.34 ± 0.11^e^	500.00^b^	1000.00^b^	8.62 ± 0.11^d^
**Gram-negative bacteria**						
*E. coli* ATCC 25922	62.50^b^	250.00^b^	19.23 ± 0.10^c^	500.00^b^	1000.00^b^	8.81 ± 0.11^b^
*P. aeruginosa* ATCC 27853	31.25^a^	125.00^a^	26.56 ± 0.2^1a^	250.00^a^	1000.00^b^	9.39 ± 0.15^a^
*E. aerogenes* ATCC 13048	31.25^a^	125.00^a^	25.47 ± 0.13^b^	250.00^a^	1000.00^b^	8.83 ± 0.14^b^
**Fungus**						
*C. albicans* ATCC 10231	62.50^b^	250.00^b^	18.86 ± 0.12^d^	500.00^b^	500.00^a^	8.74 ± 0.21^c^

The data are mean ± standard deviation. Different letters indicate significant differences (p < 0.05).

### Flow cytometric viability measurement

**Figure 1:**
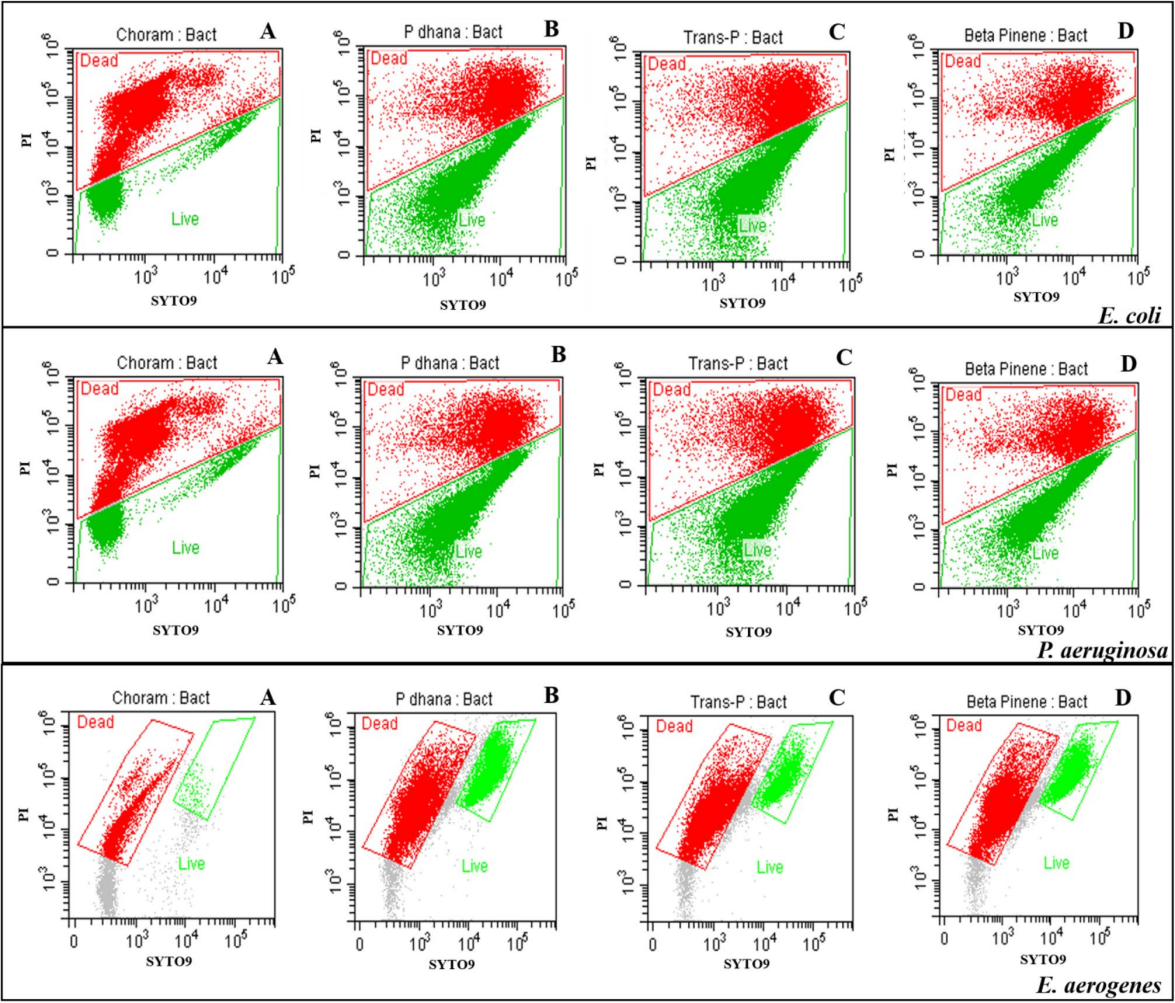
Flow cytometry dot plots of SYTO9 vs. PI of *E. coli*, *P. aeruginosa,* and *E. aerogenes* following treatment with chloramphenicol (**A**), *P. dhana* essential oil (**B**), *trans*-piperitol (**C**), and β-pinene (**D**).

**Figure 2:**
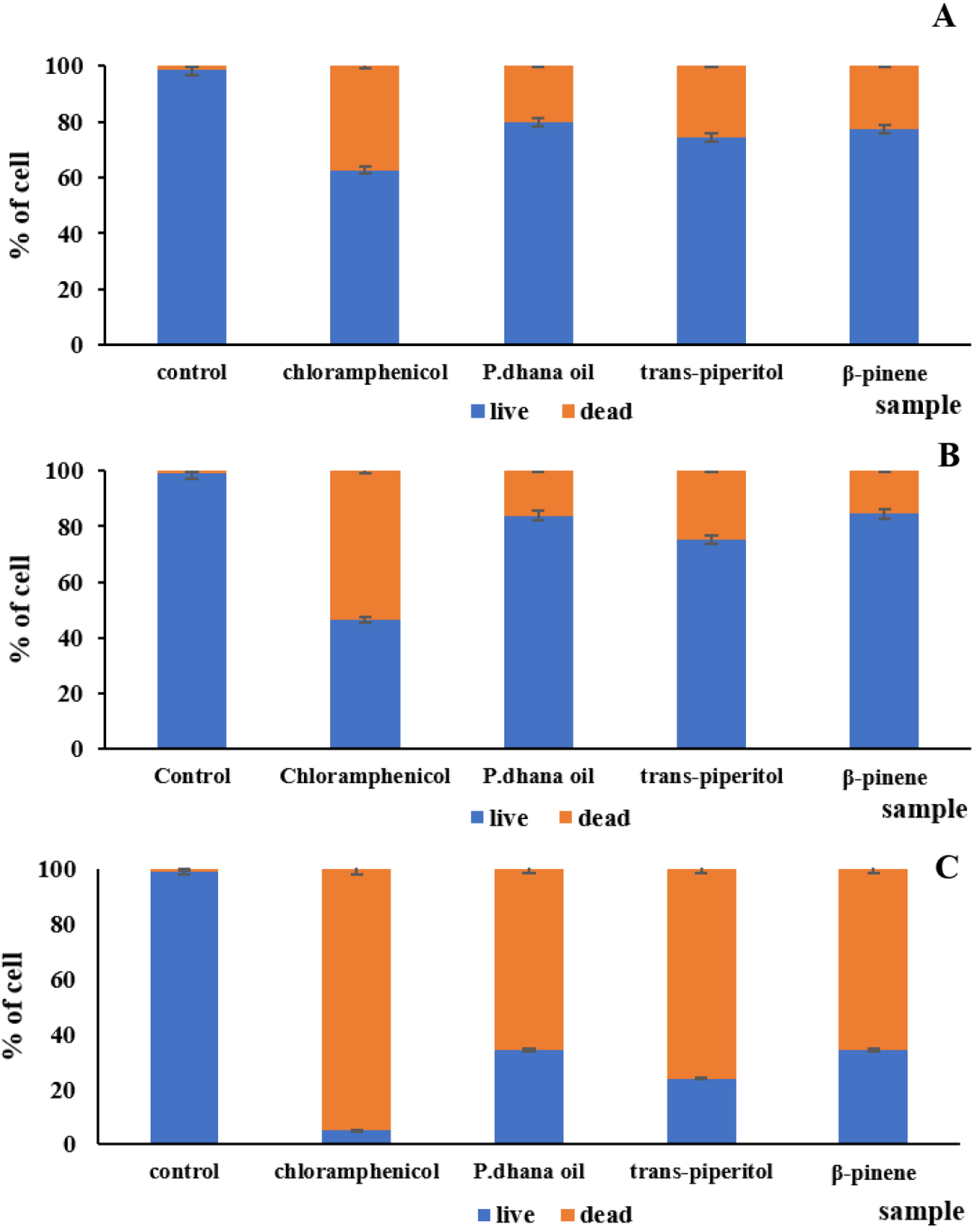
Percentages of bacterial cell subpopulations (lived cells and dead cells) of *E. coli* (**A**), *P. aeruginosa* (**B**), and *E. aerogenes* (**C**) following treatment with chloramphenicol, *P. dhana* essential oil, *trans*-piperitol, and β-pinene.

Flow cytometric analysis of membrane integrity with SYTO9/PI dual staining demonstrated a unique fluorescence pattern correlated to the degree of membrane damage. Flow cytometric plots of all treated bacteria are shown in Fig. 1. Cell membrane viability of all treated bacteria is demonstrated in Fig. 2. For *E. coli,* after a 24 h incubation, the highest percentage of dead bacteria (37.37% cell death) was seen with chloramphenicol followed by *trans*-piperitol (25.54% cell death), β-pinene (22.69% cell death), and *P. dhana* essential oil (20.14% cell death). For *P. aeruginosa,* after a 24 h incubation, the highest percentage of dead bacteria was also seen with chloramphenicol (53.56% cell death), followed by *trans*-piperitol (24.77% cell death), *P. dhana* essential oil (16.04% cell death), and β-pinene (15.34% cell death). For *E. aerogenes,* after a 24 h incubation, the highest percentage of death was seen with chloramphenicol (94.86% cell death), followed by *trans*-piperitol (76.25% cell death), *P. dhana* essential oil (65.81% cell death), and β-pinene (65.66% cell death).

### Antioxidant activity

The antioxidant activity of *P. dhana* essential oil was investigated using DPPH and ABTS-scavenging assays. Results of the antioxidant activity of *P. dhana* essential oil and Trolox are shown as IC_50_ values (Table 4). The IC_50_ values of *P. dhana* essential oil were 9.13 ± 0.11 mg/mL and 9.36 ± 0.21 mg/mL using the DPPH and ABTS assays, respectively. These values are significantly higher than those from Trolox using DPPH (0.66 ± 0.04 mg/mL) and ABTS (0.78 ± 0.04 mg/mL) assays. The antioxidant activity of *trans*-piperitol and β-pinene is also reported in Table 4. IC_50_ values of *trans*-piperitol were 14.76 ± 0.15 mg/mL and 14.98 ± 0.24 mg/mL using the DPPH and ABTS assays, respectively. Meanwhile, IC_50_ values of β-pinene were 15.45 ± 0.25 mg/mL and 15.56 ± 0.61 mg/mL using the DPPH and ABTS assays, respectively.

**Table 4:** Antioxidant activity of *P. dhana* essential oil, Trolox, *trans*-piperitol, and β-pinene.

Sample	Yield	IC_50_ (mg/mL)	
	(%w/w)	DPPH	ABTS
*P. dhana* essential oil	0.76 ± 0.21	9.13 ± 0.11^d^	9.36 ± 0.21^d^
Trolox		0.66 ± 0.04^a^	0.78 ± 0.04^a^
*trans*-Piperitol		14.76 ± 0.15^b^	14.98 ± 0.24^b^
β-Pinene		15.45 ± 0.25^c^	15.56 ± 0.61^c^

The data are mean ± standard deviation. Different letters indicate significant differences (p < 0.05).

### Cytotoxicity

The cytotoxic activity of *P. dhana* oil is shown in Table 5 as IC_50_ values. The IC_50_ values obtained from *P. dhana* essential oil were 56.63 ± 0.11 μg/mL, 51.67 ± 0.23 μg/mL, 18.24 ± 0.11 μg/mL, and 961.36 ± 0.11 μg/mL for Hela, A549, SW480, and 3T3L1 cells, respectively. Meanwhile, the IC_50_ values obtained from doxorubicin were 0.55 ± 0.21 μg/mL, 0.46 ± 0.16 μg/mL, 0.34 ± 0.13 μg/mL, and 876.34 ± 0.16 μg/mL for Hela, A549, SW480, and 3T3L1 cells, respectively. The IC_50_ values obtained from *trans*-piperitol were 7.07 ± 0.67 μg/mL, 7.76 ± 0.35 μg/mL, 65.56 ± 0.13 μg/mL, and 73.30 ± 0.23 μg/mL for Hela, A549, SW480, and 3T3L1 cells, respectively. Meanwhile IC_50_ values obtained from β-pinene were > 1000 μg/mL for all cells.

**Table 5:** Cytotoxicity of *P. dhana* essential oil, doxorubicin, *trans*-piperitol, and β-pinene.

Cell	IC_50_ (µg/mL)			
	*P. dhana* essential oil	Doxorubicin	*trans*-Piperitol	β-Pinene
Hela	56.63 ± 0.11^c^	0.55 ± 0.21^c^	7.07 ± 0.67^a^	> 1000
A549	51.67 ± 0.23^b^	0.46 ± 0.16^b^	7.76 ± 0.35^b^	> 1000
SW480	10.24 ± 0.11^a^	0.34 ± 0.13^a^	65.56 ± 0.13^c^	> 1000
3T3L1	961.36 ± 0.11^d^	876.34 ± 0.16^d^	76.30 ± 0.23^d^	> 1000

The data are mean ± standard deviation. Different letters indicate significant differences (p < 0.05).

## Discussion

Volatile compounds of the essential oils obtained from the *Peucedanum* species such as *P. longifolium*^12^, *P. ruthenicum* M. Bieb.^13,14^, *P. japonicum*^1^, *P. austriacum*^1^, *P. alsaticum*^1^, *P. paniculatum* L.^15^, *P. officinale*^16^, *P. galbanum*^1^, *P. cervaria*^1^, *P. verticillare*^1^, and *P. oreoselinum*^4^ have also been reported. For example, the main components of the essential oil obtained from the *P. longifolium* is myrcene^12^. Camphor, 1,8-cineole and sabinene are the major compounds in *P. ruthenicum* M. Bieb. essential oil^13,14^. In addition, α-pinene and β-phellandrene are the volatile compounds in *P. japonicum*, *P. austriacum*, *P. cervaria*, and *P. alsaticum* essential oils^1^. Moreover, β-cyclolavandulyl and β-isocyclolavandulyl esters are the major compounds in *P. paniculatum* L. essential oil^15^. Limonene and α-pinene are the main compounds in *P. officinale* essential oil^16^. A significant amount of p-cymene was found in *P. galbanum*, and *P. ruthenicum* M. Bieb. essential oils, while nerol was demonstrated to be the major compound in *P. verticillare* fresh fruit essential oil^1^. It is possible to detect a diversity of terpenes, especially monoterpenes, in essential oils from plant species of the *Peucedanum* genus. Factors including plant age, cultivation, climatic and micro-environmental conditions, seasons, harvest times, chemotypic variation, and geography play important roles in the various chemical compositions of essential oils from plants. For example, the amounts of γ-terpinene and β-pinene in *P. oreoselinum* fruit essential oil increased in the presence of sunlight^4^. Figuérédo et al.^16^ reported that the amount of α-phellandrene in *P. officinale* flowers essential oil was significantly higher than those obtained from other parts of the plant. Alavi et al.^13^ also reported different major compounds in the essential oils from the leaf, flower, and fruits of *P. ruthenicum* M. Bieb. In the essential oils extracted from the leaf, thymol and β-bisabolene were the major compounds. However, both compounds were not detected in essential oils extracted from the flowers and fruits. Moreover, the amount of thymol and β-bisabolene in the *P. ruthenicum* M. Bieb. leaf oil decreased as humidity increases^3^.

In our study, *P. dhana* essential oil inhibited the growth of microbial pathogens, especially Gram-negative bacteria, as evidenced by the MIC value of 62.50 µg/mL. Essential oils extracted from several species, including *P. longifolium*^12^, *ferulaefolium*^17^, *P. ruthenicum* M. Bieb.^18^, *P. japonicum*^19^, *P. alsaticum*, and *P. cervaria*^20^, had antibacterial properties. For example, the essential oil extracted from *P. ruthenicum* M. Bieb. fruits showed antibacterial activities against *S. aureus*, *S. epidermidis*, and *B. cereus*^13^. According to Pirbalouti et al.^21^, essential oil extracted from *P. membranacea* Boiss exhibited significant antibacterial activity against the *Proteus vulgaris* strain. However, there is no report on the antifungal activity from essential oil extracted from *Peucedanum* species. Our results show not only the growth reduction potential of bacterial pathogens but also antifungal activity against *C. albicans*. The antimicrobial activity of essential oils is mainly associated with the presence of active compounds, including monoterpenes, sesquiterpenes, and their derivatives, as reported by Burt^22^. Essential oils of Spanish *Mentha rotundifolia*^23^ and *Ocimum canum*^24^ are rich in piperitol and present weak antibacterial activity. In this study, *trans*-piperitol showed higher antimicrobial activity compared to that of β-pinene and the *P. dhana* essential oil. This result may depend on different pathogenic strains and concentrations of compounds. The strong antimicrobial activity may be explained by *trans*-piperitol and the synergetic effects of the major and minor compounds in the *P. dhana* essential oil^25–27^. It was found that the mode of action of the *P. dhana* essential oil may depend on the hydrophobicity of the functional groups in their chemical compounds. The hydrophobicity enables the partition of lipids of the bacterial cell membrane, which results in a disturbance of the cell wall and the cytoplasmic membrane and eventually leads to lysis and leakage of intracellular compounds^28^. In addition, Moleyar and Narasimtram^29^ stated the antimicrobial activity of volatile compounds in essential oils is a combined effect of direct and indirect vapor absorption on pathogenic strains through the aqueous medium. The vapor absorption on microbial pathogens was detected by changes in membrane permeability. The absorption into aqueous media was examined by solubility, volatility, and stability of their volatile compounds.

In addition, the impact of the *P. dhana* essential oil on the viability of bacterial cells and cell damage was estimated with highly sensitive fluorescence-based flow cytometric data. PI was used to intercalate a stain that cannot penetrate the healthy cells while SYTO9 was used to intercalate a stain that can penetrate all damaged and healthy cells. As a result, chloramphenicol, an antibiotic drug, showed higher antibacterial activity (from high level of PI with lysed cells) than other treatments. It was found that PI permeabilized cytoplasmic membranes and PI staining intercalated with DNA and directly damaged bacteria membrane. The metabolic activity of *E. coli* and *P. aeruginosa* cells was mostly weakened under the influence of *P. dhana* essential oil and other compounds compared to those found in *E. aerogenes* cells. We compared the effectiveness of two methods to determine the antibacterial effects. The results obtained from flow cytometry were not in accordance with those obtained from the resazurin assay. The observed differences between both methods suggested the presence of a sub-lethally stressed subpopulation not able to form colonies on microplates. It is additional proof that *P. dhana* essential oil in low concentrations does not cause the death of bacterial cells. Flow cytometric analysis showed that, in the presence of *P. dhana* essential oil, the observed loss of viability of bacterial strains could be correlated to membrane depolarization and membrane alteration^30^. In addition, *trans*-piperitol and β-pinene, the major compounds of *P. dhana* essential oil, possess one or several mechanisms of bacterial growth inhibition, and there may be synergetic effects among these components. These possible multiple targets and heterogenous effects could also describe the successive physiological states detected in this study in relation to membrane damage and depolarization. Our study confirmed the antimicrobial action of the *P. dhana* essential oil at the tested concentrations on bacterial cell membrane integrity.

The antioxidant activity of *P. dhana* essential oil are not yet reported. However, there are already a few reports about the antioxidant activity of essential oils obtained from other *Peucedanum* species. Normally, essential oils were evaluated as a weak antioxidant^31^. Tepe et al.^32^ found that the inhibition percentage of free radical DPPH in *P. longifolium* and *P. palimbioides* essential oils was 8.59–41.87% and 10.67–47.26%, respectively. These values are lower than those of the standard butylated hydroxytoluene and butylated hydroxyanisole compounds at 93.85% and 94.98%, respectively. Our results agreed with those from previous studies. In particular, the antioxidant activity of the *P. dhana* essential oil may be attributed to the presence of various monoterpenes like β-pinene, γ-terpinene, and limonene. β-pinene, which is a major compound in many essential oils such as turpentine oils, exhibited antioxidant activity^33^. Previous studies also confirmed that γ-terpinene and limonene exhibited good antioxidant activities. Thus, the total antioxidant activity of essential oil may be correlated to the antioxidant activities from the volatile compounds listed in Table 1, and not only from the major ones that have low antioxidant activity. The DPPH and ABTS assays are hydrogen atom abstraction assays. The radical scavenging abilities of chemical compounds in essential oils may be related to the bond dissociation energies (BDEs) of the compounds^34^. In the case of γ-terpinene, low BDE was measured due to the abstraction of three potential allylic hydrogen atoms. As a result, hydrogens in the ring have much lower BDEs than isopropyl hydrogen. However, antioxidant activities of essential oils or pure compounds have shown less correlation between DPPH and ABTS activities. DPPH and ABTS assays were considered as complicated thermodynamic assays based on kinetic and stoichiometric effects and may also be related to complex multistep reaction mechanisms^34^.

Due to the cytotoxicity of essential oils, they have been applied as potential antitumor agents. This study evaluated the cytotoxicity of essential oil extracted from *P. dhana* fruits in both cancer and normal cells using an MTT assay. The *P. dhana* essential oil, *trans*-piperitol, β-pinene, and the positive control, doxorubicin demonstrated different cytotoxicity depending on the cells. Varied results may be related to the functional groups represented in each compound or composition. However, IC_50_ values obtained from antimicrobial activity tests of *P. dhana* essential oil, suggest that the *P. dhana* oil has a moderate to strong cytotoxic effect. This result agrees with those from previous studies on essential oils from various plant species. Sylvestre et al.^35^ reported the IC_50_ values of essential oils and their corresponding cytotoxic activity. IC_50_ values of 10–50 μg/mL, 50–100 μg/mL, 100–200 μg/mL, and 200–300 μg/mL indicate strong, moderate, weak, and very weak cytotoxic properties. In addition, IC_50_ values higher than 300 μg/mL indicate no cytotoxicity. Considering the cytotoxic activity on the four cells tested with the MTT assay, *P. dhana* essential oil showed moderate cytotoxic activity against the Hela and A549 cells, and strong cytotoxic activity against SW480 cells. The doxorubicin, used as a positive control in this study, had strong cytotoxic activity against cancer cells. Doxorubin is considered a broad-spectrum antitumor antibiotic and is most extensively used in chemotherapy regimens for cancer patients. β-pinene, α-pinene, and γ-terpinene are considered to be the compounds responsible for some of the cytotoxic activities^36,37^. However, other compounds such as limonene, β-cubebene, α-terpineol, and camphene are previously known to have cytotoxic effects against different cells^38^. The molecular mechanism of the cytotoxicity of *P. dhana* essential oil may be the induction of apoptosis and necrosis. In eukaryotic cells, essential oils can induce depolarization of the mitochondrial membranes, thereby decreasing the pH gradient and affecting calcium, other ions, the proton pump, and the ATP pool^39^. Membrane fluidity also changed and became abnormally permeable, demonstrating the leakage of radicals, cytochrome c, calcium ions, and proteins. The permeabilization of the inner and outer mitochondrial membranes resulted in cell death by apoptosis and necrosis. Considering an investigation about the cytotoxic effect of *Peucedanum* spp. on tumor cells, Yeong et al.^39^ reported that the *P. japonicum* Thunb essential oil revealed cytotoxic activity against A549 cells with an IC_50_ of 0.04192% v/v. The results obtained from this study also present the importance of *P. dhana* as an alternative herbal source of essential oil that can be used as a cytotoxic agent against cancer cells, especially in human colonic adenocarcinoma.

## Conclusion

The major volatile compounds of the essential oil obtained from *P. dhana* fruits were *trans*-piperitol, β-pinene, and o-cymene. The *P. dhana* essential oil had strong antimicrobial activity against Gram-negative bacteria, Gram-positive bacteria, and the fungus *C. albicans*. It was demonstrated using flow cytometry and specific staining that the *P. dhana* essential oil has bacteriostatic action in vitro against test bacteria at MIC, with combined effects on cell membrane alteration and depolarization. The *P. dhana* essential oil may not directly damage the bacterial outer membrane due to different dynamics of bacterial viability states from staining results. The essential oil showed antioxidant activity mainly through the inhibition of DPPH and ABTS assays and had cytotoxic effects against the tested cancer cells, especially the SW480 cells. The strong biological activity of *P. dhana* essential oil may be due to *trans*-piperitol and the synergistic interactions of the terpenes. Our results highlight the potential of *P. dhana* as a source of essential oil for pharmaceutical applications, such as antimicrobials and health promoters.

## Materials and methods

### Chemicals and media

Anhydrous sodium sulfate, sodium carbonate, dichloromethane, chloramphenicol, β-pinene dimethylsulfoxide, DPPH, Trolox, methanol, ABTS, potassium persulfate, gallic acid, Folin-Ciocalteu reagent, gallic acid, MTT, and resazurin were purchased from Sigma-Aldrich (USA). *Trans*-piperitol was purchased from BioCrick (China). Dulbecco's Modified Eagle medium (DMEM) and fetal bovine serum were purchased from Gibco (USA). Sabouraud dextrose and Müller-Hinton broths were purchased from Becton, Dickinson, and Company (USA) and YM (USA), respectively.

### Plant material

*P. dhana* A. Ham fruits were collected during their flowering stage at Thung Hua, Wang Nuea district, located in Lampang province, Northern Thailand (latitude: 19° 23′ 75.3″ S, longitude: 99° 57′ 50.6″ W, altitude: 720 m) in December 2019. The collection site access was approved by Mr. Vichien Tammasorn, the farm owner. The plant was identified by taxonomist Dr. Jantrararuk Tovaranonte, head of Mae Fah Luang Botanical Garden and a voucher specimen (No. 10124) was deposited in the Mae Fah Luang Botanical Garden, Mae Fah Luang University, Chiang Rai, Thailand. Harvested plant material was dried indoors at room temperature for 2 weeks and further stored in plastic containers at room temperature until use. This study complies with relevant institutional, national, and international guidelines and legislation.

### Essential oil extraction

The dried *P. dhana* fruits (500 g) were subjected to hydrodistillation for 4 h using a Clevenger-type apparatus (Duran West Germany). The obtained fruit essential oil was collected and combined with anhydrous sodium sulfate to remove the water. The essential oil was collected in a vial, sealed, and stored at 4 °C. The essential oil yield was calculated as [mass of essential oil obtained (g)/mass of dry sample (g)] × 100. This extraction was performed three times.

### Identification of chemical composition by gas chromatography-mass spectrometry (GC–MS)

First, the essential oil was diluted with dichloromethane (1:100 v/v). The chemical composition of the *P. dhana* essential oil was identified using an Agilent 6890 N gas chromatograph connected to a mass spectrometer (Agilent 5973 network mass selective detector, Agilent Technologies, Santa Clara, CA, USA). A fused-silica capillary DB5-MS (30 m × 0.25 mm i.d., 0.25 μm) (J&W Scientific, USA) was used in the system. A total of 1 μL of sample was injected using a split ratio of 1:50. The oven temperature started at 60 °C, then increased to 240 °C at a rate of 3 °C/min. Helium (99.99% purity) was used as the carrier gas with a flow rate of 1 mL/min. Electron impact ionization was used, with the electron energy set to 70 eV. The ion source temperature was set to 250 °C. The acquisition was performed in scan mode (*m/z* 30–300). Quantitative analysis was performed based on the total ion count detected by the GC–MS. Compounds were identified using their retention indices and mass spectra. Retention indices were calculated using linear interpolation of the retention times of C_9_–C_17_ *n*-alkanes on both the DB-1 (100% dimethylpolysiloxane) and DB5-MS (5% diphenyl/95% dimethylpolysiloxane) column (30 m × 0.25 mm i.d., 0.25 μm) (J&W Scientific, USA) as well as further compared to corresponding reference standard data reported by Adams^10^, Babushok et al.^11^, and the mass spectra from the W8N08 and Wiley 7 N libraries. Quantitative analysis of volatile compounds was performed using a relative peak area percentage on the spectra taken using an Agilent 6890 N gas chromatograph connected to a flame ionization detector (Agilent Technologies, Santa Clara, CA, USA). The same parameters as those used to identify the essential oil were used. The injector and detector temperatures were 250 °C and 280 °C, respectively. The experiment was performed three times.

### Antimicrobial activity

#### Microbial strains

Seven human pathogens were used in this study including three Gram-positive bacteria: *Staphylococcus aureus* ATCC 25923, *S. epidermidis* ATCC 12228, and *Bacillus cereus* ATCC 11778, three Gram-negative bacteria: *Escherichia coli* ATCC 25922, *Pseudomonas aeruginosa* ATCC 27853, and *Enterobacter aerogenes* ATCC 13048, and one fungus: *Candida albicans* ATCC 10231. All bacterial strains were obtained from the Department of Medical Science, Ministry of Health, Bangkok, Thailand while the fungus *C. albicans* ATCC10231 was obtained from the culture collection of the Faculty of Dentistry, Khon Kaen University.

#### Disc diffusion test

The antimicrobial screening was conducted by a modified disc diffusion method^27^. All bacterial strains were sub-cultured in Müller–Hinton broth while the fungus was sub-cultured in sabouraud dextrose broth. All strains were incubated at 37 °C for 24 h. The turbidity of the cell suspension was measured at 600 nm and adjusted with broth media to reach a 0.5 McFarland standard. The *P. dhana* essential oil, chloramphenicol, *trans*-piperitol, and β-pinene were prepared at a concentration of 1000 µg/mL in 10% dimethylsulfoxide (DMSO) and sterilized distilled water, respectively. Each bacterial strain was spread on a sterile Petri dish containing Müller–Hinton or sabouraud dextrose agar using a sterile cotton swab. Then, 30 μL of each sample was dropped on a 6 mm diameter sterilized paper disc (Whatman, USA) and placed on a Petri dish. All bacterial plates were incubated at 37 °C for 24 h, while fungal plates were incubated at 28 °C for 48 h. The diameter of the inhibition clear zone after incubation was measured in millimetres by a Vernier caliper. 10% DMSO and chloramphenicol were used as negative and positive controls in the same conditions as essential oil, respectively. Each experiment was carried out in triplicate.

#### MIC and MMC analysis

MIC values inhibiting the growth of tested pathogens were measured by a broth microdilution method according to a modified method^27^. The essential oil, chloramphenicol, *trans*-piperitol, and β-pinene at different concentrations were dissolved in 10% DMSO and sterilized distilled water with the following concentrations, respectively: 1000, 500, 250, 125, 62.50, 31.25, 15.62, and 7.81 µg/mL. A two-fold dilution procedure was used for all mixtures. The experiment was performed in sterile 96-well microtiter plates. A combination of 10 μL of microbial suspension (10^6^ CFU/mL) of each pathogen and 0.675% resazurin were added to each well containing 50 µL of sample. Müller–Hinton medium was used for bacteria, while sabouraud dextrose medium was used for the fungus. All plates were covered with a sterile cap. The bacterial plates were incubated at 37 °C for 4 h, while fungal plates were incubated at 28 °C for 4 h. 10% DMSO and chloramphenicol served as negative and positive controls, respectively. The MIC was determined by a change in color. The color of each well was compared to those obtained from the negative control. The lowest concentration with the same color as the negative control was interpreted as the MIC (absence of turbidity). All bacterial plates were incubated at 37 °C for 24 h while fungal plates were incubated at 28 °C for 48 h. The colony formation was detected after incubation. The lowest concentration of essential oil sample that was able to effectively reduce the growth of microorganisms (99.5%) on the agar medium was determined as the MMC.

### Flow cytometry

According to the lowest MIC of *P. dhana* essential oil for *E. coli, P. aeruginosa,* and *E. aerogenes* strains, the intracellular metabolic activity of these three strains was investigated by measurement of the redox potential using a flow cytometer with a modified method by Girard et al.^40^ Flow cytometric analysis was performed on a DxFLEX Flow Cytometer (BeckmanCoulter Inc., Miami, FL, USA). Data were acquired using light-scatter and fluorescence signals resulting from 15 mW laser illumination at 488 nm. In total, 1 mL of Müller-Hinton broth medium was inoculated with bacterial suspension (10^6^ CFU/mL) and supplemented individually with *P. dhana* essential oil, *trans*-piperitol, β-pinene, and chloramphenicol at the MIC concentration. A control sample was similarly prepared without any essential oil, standard, or drug. Cytometric analysis was carried out after a 24 h incubation of the bacteria at 37 °C with *P. dhana* essential oil, *trans*-piperitol, β-pinene, and chloramphenicol. The solution was removed and the probes were centrifuged at 5000 rpm for 5 min. The bacterial pellet was diluted with 250 µL of 70% isopropanol. Cells were stained with 1.5 µL of PI and 1.5 µL of SYTO9 from a commercial BacLight TM kit (Live/Dead Bacterial viability BacLight TM kit, Thermo Fischer Scientific, Waltham, MA, USA). After 10 min of incubation at room temperature (27 °C) without light, the cells were analyzed. Each sample was analyzed in triplicate. Light-scatter and fluorescence measurements were acquired logarithmically and data were analyzed with CytExpert for DxFLEX software (BeckmanCoulter Inc., Miami, FL, USA). The analysis of the fluorescence signals from both fluorochromes preceded a doublets discrimination procedure using height versus width scatter signals measurement in order to discriminate single events from conglomerates. The populations were then defined by gating in the dot plots of green fluorescence (SYTO9) versus red fluorescence (PI).

### Antioxidant activity

#### DPPH assay

The scavenging capacity against DPPH of the *P. dhana* essential oil was evaluated according to the modified method^34^. The essential oil, *trans*-piperitol, β-pinene, and a standard reference, T, were prepared in methanol at the following concentrations: 1000, 500, 250, 125, 62.50, 31.25, 15.62, and 7.81 µg/mL. Two-fold dilution was employed for each mixture. 0.05 mL of each essential oil and Trolox were mixed with 1.95 mL of 0.2 mol/L methanolic solution of DPPH. The mixture was shaken vigorously and kept in the dark at 27 °C for 30 min. The absorbance of the mixture was determined at 517 nm using a PerkinElmer spectrophotometer. Methanol was used as a blank solution. The scavenging capacity was calculated as [(A_C_ − A_s_/A_c_) × 100 where A_C_ and A_S_ correspond to the absorbance of the control and sample, respectively. The antioxidant activity of *P. dhana* essential oil and Trolox by DPPH assay was reported as IC_50_. Each sample was tested for antioxidant activity in triplicate.

#### ABTS assay

The scavenging activity against ABTS of the *P. dhana* essential oil was determined according to the modified method^34^. The ABTS radical cation was prepared by mixing 7 mM ABTS solution with 2.45 mM potassium persulfate and kept in the dark at 27 °C. The same concentrations of essential oil, *trans*-piperitol, β-pinene, and Trolox were prepared as described in the DPPH assay. For each concentration, 50 mL of each sample prepared in methanol was mixed with 150 mL of ABTS radical solution before being shaken vigorously and kept in the dark at 27 °C for 5 min. The absorbance of the solution was determined at 734 nm using a UV/Vis spectrophotometer (PerkinElmer, USA). Methanol was used as a blank solution. Trolox was also used as a standard reference. The scavenging capacity was calculated using the equation described in the DPPH assay. Trolox was used as a standard reference. The antioxidant activity of *P. dhana* essential oil and Trolox by the ABTS assay was also reported as IC_50_. Each sample was tested for antioxidant activity in triplicate.

### In vitro cytotoxicity activity

#### Cell and culture

The SW480, A549, Hela cells, and 3T3L1 cells were purchased from the China Center for Type Culture Collection. These cells were cultured in Dulbecco's Modified Eagle medium supplemented with 10% fetal bovine serum and incubated in a humidified incubator at 37 °C and 5% CO_2_ humidified atmosphere in an incubator (Shel lab CO_2_ Series, USA).

#### MTT assay

The cytotoxicity of the *P. dhana* essential oil was evaluated by using the MTT reduction inhibition assay^38^. Briefly, the cells were grown in 96-well culture plates at a density of 1 × 10^4^ cells/well in 1 mL of fresh medium. After 24 h, cells were treated with different concentrations of essential oil, *trans*-piperitol, and β-pinene (1000, 500, 250, 125, 62.50, 31.25, 15.62, and 7.81 µg/mL) in culture plates. The plates were incubated for 24 h. DMSO treated cells were used as controls. The medium was aspirated, the MTT solution (10 μL of 5 mg/mL in stock solution) was added to each well, and was further incubated for 4 h. After incubation, 200 μL of DMSO was added to dissolve the formed product prior to being placed in a shaking incubator for 10 min. The blue dissolved formazan crystals (200 µL) were transferred to a 96-well plate and measured at 570 nm using a multi-scan microplate reader (BioTek Instruments, Inc.). The control consisted of treating cells with DMSO for the same duration of time. Doxorubicin was used as a positive control. The cytotoxic activity of *P. dhana* oil against all cells was reported as IC_50_ values. The mean IC_50_ values were calculated by a non-linear regression using version 5.0 of the Graphpad Prism software for Windows (GraphPad Software, USA).

### Statistical analysis

Results are expressed as the mean ± standard deviation (SD). All experiments were performed in triplicate. Analysis of variance (ANOVA) was performed to compare the antimicrobial activities of the *P. dhana* essential oil and its major components. ANOVA was also used to compare the antioxidant activities, and cytotoxic activities of the *P. dhana* essential oil against specific compounds. The mean comparison was based on Student's t-test at p < 0.05. All statistical tests were performed using SPSS statistics software (IBM SPSS Statistics for Windows, Version 22.0. Armonk, NY, IBM Corp).
